# Supplementary material for: Vegetables and fruits retailers in two urban areas of Bangladesh: Disruption due to COVID– 19 and implications for NCDs
Source: PLoS One. 2023 Jan 10;18(1):e0280188. doi: 10.1371/journal.pone.0280188 (PMC9831295; doi:10.1371/journal.pone.0280188)
Supplement: S1 Table — (DOCX) [file pone.0280188.s001.docx]

**Table S1: Mapping of Vegetables and Fruits POS Retailers and Markets in Dhaka and Manikganj**

| **District** | **Thana** | **Total Market** | **SUP** | **WM** | | | **SS** | | | **MV** | | | **Total** |
| --- | --- | --- | --- | --- | --- | --- | --- | --- | --- | --- | --- | --- | --- |
|  |  |  |  | **V** | **F** | **V&F** | **V** | **F** | **V&F** | **V** | **F** | **V&F** |  |
| Dhaka | Pallabi | 13 | 7 | 171 | 52 | 16 | 51 | 43 | 1 | 163 | 113 | 0 | 610 |
|  | Badda | 20 | 4 | 225 | 62 | 13 | 89 | 93 | 6 | 94 | 63 | 4 | 649 |
|  | Tajgaon | 16 | 2 | 504 | 258 | 2 | 48 | 42 | 3 | 51 | 34 | 0 | 942 |
|  | Uttara | 10 | 11 | 95 | 95 | 13 | 32 | 91 | 0 | 53 | 54 | 0 | 433 |
|  | Lalbag | 7 | 3 | 146 | 36 | 0 | 14 | 2 | 0 | 35 | 23 | 0 | 256 |
|  | Mohammadpur | 15 | 3 | 276 | 82 | 0 | 27 | 31 | 0 | 92 | 68 | 0 | 576 |
|  | Total | 81 | 30 | 1417 | 585 | 44 | 261 | 302 | 10 | 488 | 355 | 4 | 3466 |
|  |  |  |  |  |  |  |  |  |  |  |  |  |  |
| Manikganj | Manikganj Sadar | 10 | 0 | 126 | 113 | 5 | 94 | 65 | 8 | 0 | 4 | 0 | 415 |
|  | Singair | 6 | 0 | 105 | 47 | 0 | 48 | 15 | 2 | 0 | 1 | 0 | 218 |
|  | Total | 16 | 0 | 231 | 160 | 5 | 142 | 80 | 10 | 0 | 5 | 0 | 633 |

**Note: Here WM indicates “Wet Market”, SUP indicates “Super Shop”, SS indicates “Street Shops” and MV indicates “Mobile Vendors”**
