# Supplementary material for: Vegetables and fruits retailers in two urban areas of Bangladesh: Disruption due to COVID– 19 and implications for NCDs
Source: PLoS One. 2023 Jan 10;18(1):e0280188. doi: 10.1371/journal.pone.0280188 (PMC9831295; doi:10.1371/journal.pone.0280188)
Supplement: S2 Table — (DOCX) [file pone.0280188.s002.docx]

**Table S2: Socio-demographic and business profile of the vegetables and fruits retailers**

| **Socio Demographic and Business Profile** | | **Frequency**  **(n=1319)** | **Percentage (%)** |
| --- | --- | --- | --- |
| Gender | Male | 1289 | 97.73 |
|  | Female | 30 | 2.27 |
| Educational Qualification | No Formal Education | 438 | 33.21 |
|  | Primary Education | 544 | 41.24 |
|  | Secondary Education | 275 | 20.85 |
|  | Higher Secondary Education | 49 | 3.71 |
|  | Graduate/Post Graduation Education | 13 | 0.99 |
| Wealth Group | Very Low | 283 | 21.46 |
|  | Low | 269 | 20.39 |
|  | Medium | 332 | 25.17 |
|  | High | 376 | 28.51 |
|  | Very High | 59 | 4.47 |
| Age of the vendors | 11 – 30 years | 361 | 27.37 |
|  | 31 – 40 years | 454 | 34.42 |
|  | 41 – 50 years | 335 | 25.4 |
|  | 51 – 60 years | 124 | 9.4 |
|  | 61 years and above | 45 | 3.42 |
| Duration of Business (Years) | 0 – 5 years | 476 | 36.09 |
|  | 6 – 10 years | 414 | 31.39 |
|  | 11 – 15 years | 228 | 17.29 |
|  | 16 years and above | 201 | 15.24 |
| Primary amount of investment  (In Bangladeshi taka) | Below 10,000 | 672 | 50.95 |
|  | 10,001 - 30,000 | 390 | 29.57 |
|  | 30,001 - 50,000 | 154 | 11.68 |
|  | 50,001 - 1,00,000 | 68 | 5.16 |
|  | 1,00,001 and above | 35 | 2.65 |
| Source of funds  (Multiple responses available) | Self-funding | 1219 | 92.42 |
|  | Friends and family | 466 | 35.33 |
|  | Loan from local money lenders | 96 | 7.28 |
|  | NGO/ Bank or financial institutions and Others | 112 | 8.29 |
| Type of sellers | Mobile vendor | 298 | 22.59 |
|  | Street vendor | 470 | 35.63 |
|  | Wet market retailer | 551 | 41.77 |
| Type of products | Sell only vegetables | 847 | 64.22 |
|  | Sell only fruits | 453 | 34.34 |
|  | Sell fruits, vegetables and others | 19 | 1.44 |
| Number of staffs | Self-supported/self-managed | 1003 | 76.04 |
|  | One staff | 227 | 17.21 |
|  | Two staffs | 60 | 4.55 |
|  | Three and more | 29 | 2.2 |
