# Supplementary figures and images for: Vegetables and fruits retailers in two urban areas of Bangladesh: Disruption due to COVID– 19 and implications for NCDs
Source: PLoS One. 2023 Jan 10;18(1):e0280188. doi: 10.1371/journal.pone.0280188 (PMC9831295; doi:10.1371/journal.pone.0280188)

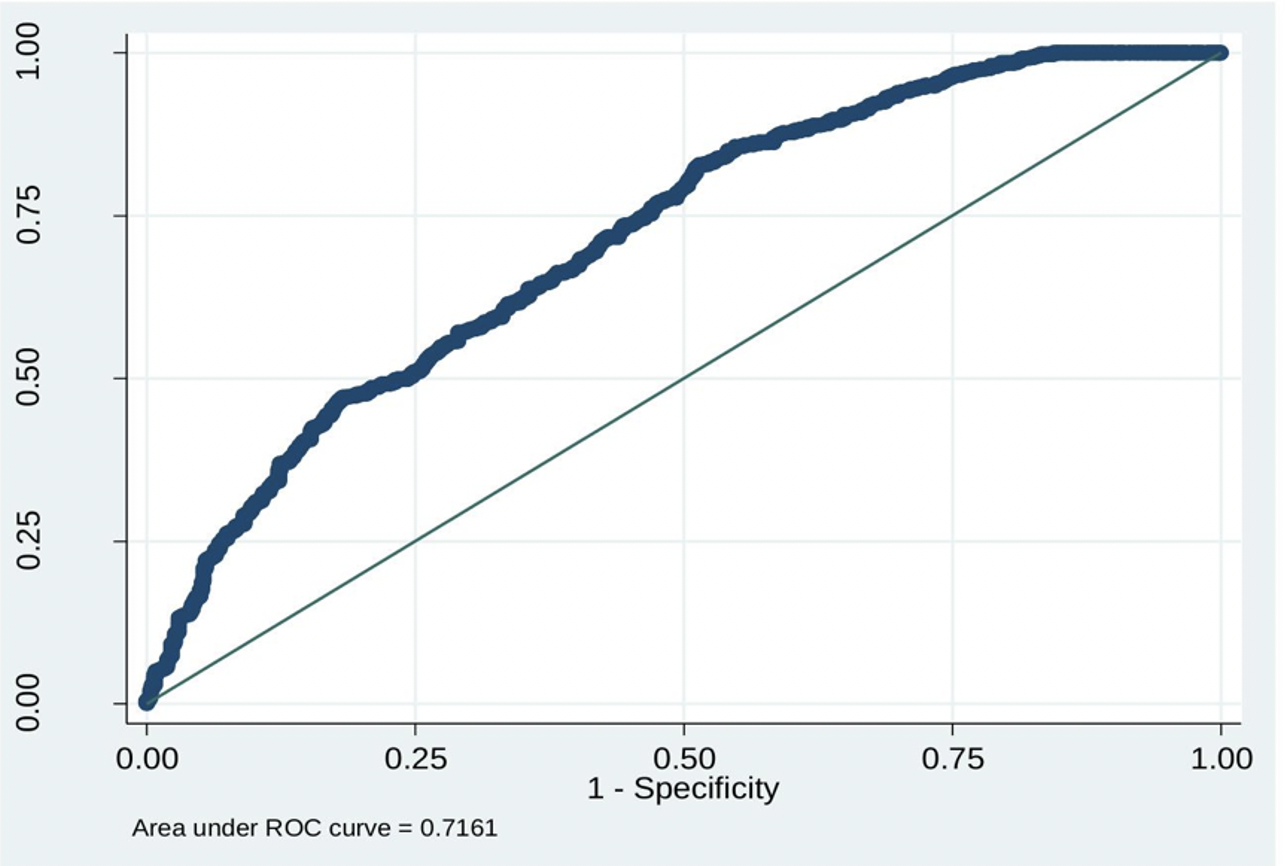

Supplement: S1 Fig — (TIF) [file pone.0280188.s004.tif]
