# Supplementary material for: Vegetables and fruits retailers in two urban areas of Bangladesh: Disruption due to COVID– 19 and implications for NCDs
Source: PLoS One. 2023 Jan 10;18(1):e0280188. doi: 10.1371/journal.pone.0280188 (PMC9831295; doi:10.1371/journal.pone.0280188)
Supplement: Attachment — Submitted filename: Reviewers Evaluation Report of Manuscript # PONE-D-22-20793.docx [file pone.0280188.s005.docx]

**Review of Manuscript: Vegetables and fruits retailers in two urban areas of Bangladesh: Disruption due to COVID – 19 and implications for NCDs**

***Manuscript ID: PONE-D-22-20793***

***Review Status:***

|  | ***Comments/Remarks*** |
| --- | --- |
| ***Summary*** | A study of 1,319 retailers was undertaken in two urban regions, namely Dhaka and Manikganj, between September 2021 and October 2021 with the goal of determining the influence of COVID-19 on the business practices and outcomes for the vegetables and fruit retailers in Bangladesh.  After review the paper it has been identified that the paper is needed revision to publish in *‘PLOS ONE’*. The review report is given below: - |
| ***Title*** | Impact of COVID-19, the second part of the paper's title, is preferable to having it in just one section. |
| ***Abstract*** | The abstract needs to follow the summary of the Background, Methods, Findings, and Interpretation. |
| ***Introduction*** | The rationale for the study is explained in the introduction section, but the study's gap with respect to prior research, contribution to the global context, and guidance throughout the paper are all absent. |
| ***Research Methodology*** | If the sample had been a bit stronger, the base would have been more robust. It might be able to uncover more precise explanations for the retailers, for instance, if certain rural and urban parts of the nation were compared and improved. |
| ***Analysis of Empirical Results*** | The results are presented in a proper and understandable way. The findings and the results are consistent. |
| ***Discussion*** | The authors incorporate all of the discussion based on the empirical data, although it must be evaluated in light of earlier work. |
| ***Conclusion*** | Writing and thoroughly outlining the policy implications in accordance with research in Bangladeshi contexts will provide more value. |
| ***Tables and Figures*** | Tables and Figures need to reconfirmed according to the author’s guidelines of *‘PLoS ONE’.* |
| ***References*** | It is highly recommended that referencing styles should be reconfirmed according to the author’s guidelines of *‘PLoS ONE’.* |
| ***Others/Overall*** | This manuscript has met the quality requirements for publication in *'PLoS ONE'* I wish the writers continued success in their efforts to improve the paper. |
